# Rebleeding of Ruptured Intracranial Aneurysm After Admission: A Multidimensional Nomogram Model to Risk Assessment

**DOI:** 10.3389/fnagi.2021.692615

**Published:** 2021-09-01

**Authors:** Qingyuan Liu, Yi Yang, Junhua Yang, Maogui Li, Shuzhe Yang, Nuochuan Wang, Jun Wu, Pengjun Jiang, Shuo Wang

**Affiliations:** ^1^Department of Neurosurgery, Beijing Tiantan Hospital, Capital Medical University, Beijing, China; ^2^Department of Blood Transfusion, Beijing Tiantan Hospital, Capital Medical University, Beijing, China; ^3^China National Clinical Research Center for Neurological Diseases, Beijing, China

**Keywords:** ruptured intracranial aneurysms, rebleeding, morphology, hemodynamics, multidimensional predicting model

## Abstract

**Objective:**

Rebleeding is recognized as the main cause of mortality after intracranial aneurysm rupture. Though timely intervention can prevent poor prognosis, there is no agreement on the surgical priority and choosing medical treatment for a short period after rupture. The aim of this study was to investigate the risk factors related to the rebleeding after admission and establish predicting models for better clinical decision-making.

**Methods:**

The patients with ruptured intracranial aneurysms (RIAs) between January 2018 and September 2020 were reviewed. All patients fell to the primary and the validation cohort by January 2020. The hemodynamic parameters were determined through the computational fluid dynamics simulation. Cox regression analysis was conducted to identify the risk factors of rebleeding. Based on the independent risk factors, nomogram models were built, and their predicting accuracy was assessed by using the area under the curves (AUCs).

**Result:**

A total of 577 patients with RIAs were enrolled in this present study, 86 patients of them were identified as undergoing rebleeding after admission. Thirteen parameters were identified as significantly different between stable and rebleeding aneurysms in the primary cohort. Cox regression analysis demonstrated that six parameters, including hypertension [hazard ratio (HR), 2.54; *P* = 0.044], bifurcation site (HR, 1.95; *P* = 0.013), irregular shape (HR, 4.22; *P* = 0.002), aspect ratio (HR, 12.91; *P* < 0.001), normalized wall shear stress average (HR, 0.16; *P* = 0.002), and oscillatory stress index (HR, 1.14; *P* < 0.001) were independent risk factors related to the rebleeding after admission. Two nomograms were established, the nomogram including clinical, morphological, and hemodynamic features (CMH nomogram) had the highest predicting accuracy (AUC, 0.92), followed by the nomogram including clinical and morphological features (CM nomogram; AUC, 0.83), ELAPSS score (AUC, 0.61), and PHASES score (AUC, 0.54). The calibration curve for the probability of rebleeding showed good agreement between prediction by nomograms and actual observation. In the validation cohort, the discrimination of the CMH nomogram was superior to the other models (AUC, 0.93 vs. 0.86, 0.71 and 0.48).

**Conclusion:**

We presented two nomogram models, named CMH nomogram and CM nomogram, which could assist in identifying the RIAs with high risk of rebleeding.

## Introduction

Intracranial aneurysms (IAs), a common cerebrovascular disease in the aging population, refer to the main cause of subarachnoid hemorrhage. Rebleeding is recognized as a catastrophic event with high mortality after aneurysmal subarachnoid hemorrhage (aSAH) (Rosenørn et al., 1987; Jaechan et al., 2015; Kienzler et al., 2016). Though timely surgical intervention can effectively protect aSAH patients from poor outcome (Ko et al., 2011; Cordonnier et al., 2018; Darkwah Oppong et al., 2018), for several reasons, a notable number of patients cannot receive treatment as soon as they are sent to a hospital. For the reason that the most rebleeding occurs within 6 h after the initial hemorrhage (Rosenørn et al., 1987; Hijdra et al., 1988; Jaechan et al., 2015), patients to be prioritized should be determined.

The key to making medical decisions for this condition is to identify the rebleeding risk of ruptured intracranial aneurysms (RIAs). However, a predicting model has not been built, or reliable factors have not been set to discriminate the RIAs at high risk of rebleeding. There are several aspects involved in the mechanism of IAs rupture, which primarily include the structure damage of the aneurysm wall (Frösen et al., 2012) and the hemodynamic condition of IAs (Meng et al., 2012, 2014; Dolan et al., 2013). Though some comorbidities could elevate the risk of rebleeding (e.g., hypertension), rebleeding still occurred in approximately 22% of patients after they had received the effective management (Boogaarts et al., 2015). It is noteworthy that some existing studies reported that morphological characteristics could predict the risk of rebleeding after aSAH (Starke et al., 2011; Boogaarts et al., 2015). However, a meta-analysis revealed the low quality of current evidence and low predicting accuracy of reported parameters (Boogaarts et al., 2015), which may be insufficient in clinical risk assessment. As indicated from our preliminary study, the hemodynamic characteristics could help discriminate the RIAs at high risk of rebleeding (Liu et al., 2019). Based on the mentioned facts, this study assumed that building a multidimensional predicting model can effectively discriminate the RIAs at high risk of rebleeding.

The present study aimed to build a risk assessment model by exploiting multidimensional characteristics of RIAs. The clinical, morphological, and hemodynamic characteristics of a group of RIAs in a neurosurgical center were retrospectively reviewed. This study considered that this current can present more insights into the factors of rebleeding and contribute to better medical decisions.

## Materials and Methods

### Patient Selected and Study Design

The patients with RIAs from January 2018 to September 2020 were retrospectively reviewed. Patients were enrolled by complying with the following standards: (1) an angiogram (CT angiogram, CTA) was performed after IA rupture; (3) the patients were sent to our institution within 12 h as soon as aSAH was identified (by symptoms, e.g., acute headache and sudden coma); and (4) clinical records were complete, or clinical history can be traced.

This study excluded the patients (1) having other intracranial tumors, angiostenosis and angio-malformation (e.g., arteriovenous malformation and cavernous malformation); (2) having a family history of IAs or connective tissue disease; (3) having multiple IAs, causing the source of the bleeding or rebleeding difficult to identify; (4) with dissecting or thrombus IAs; and (5) receiving special treatment for RIAs in other medical institutions before admission.

Rebleeding was the primary endpoint in this study and was diagnosed based on radiological findings: the magnitude of subarachnoid, intracerebral, or intraventricular blood significantly increased on CT after the admission, and the magnitude of bleeding did not increase and remained stable at/before admission.

The IAs which underwent rebleeding after the admission were identified as the rebleeding aneurysms, whereas the IAs without rebleeding before surgical intervention were found as the stable aneurysms. Rebleeding events were identified by two experienced neurosurgeons (PJ and JW, who were blind to clinical information and had worked as cerebral vascular neurosurgeons for more than 5 years) in accordance with the bleeding presentation on medical record and CT after the admission. Furthermore, the discrepancies were solved by consulting a senior neurosurgeon (SW, who had worked as a cerebral vascular neurosurgeon for more than 15 years).

Patients enrolled from August 2018 to December 2019 formed the primary cohort (411 patients with 411 IAs), which was adopted to develop the predictive model; while patients enrolled from January 2020 to September 2020 were classified as the validation cohort (127 patients with 127 IAs). The ratio of numbers of unruptured IAs in the primary cohort and the validation cohort reached approximately 3:1.

### Perioperative Management

After admission, acute lowering of systolic pressure to 120–140 mmHg was the target (Connolly et al., 2012; American Society of Anesthesiologists Task Force on Perioperative Blood Management., 2015); all patients with Hunt-Hess I-II received surgical intervention within 72 h. However, once neurological condition progressively deteriorated, an emergency intervention would be performed.

Specific to patients with Hunt-Hess grade III-V at the admission, immediate surgical intervention was not recommended (Connolly et al., 2012). The patients who had not received immediate intervention would receive standard care following the guidelines (Connolly et al., 2012). After the admission, a CT would be performed per day, or when the patient had a sudden disorder of consciousness, or gradually worsening neurological states or convulsion after the admission. Surgical intervention was only considered when the patient’s neurological status progressively deteriorated, or a rebleeding or a cerebral hernia was found in the radiological examination.

### Clinical Information and Morphology Assessment

Clinical information was collected from electronical medical records about age, gender, comorbidities (e.g., hypertension, dyslipidemia, diabetes mellitus, coronary heart disease, and ischemic stroke), Hunt-Hess grade at the admission, time from the admission to the rebleeding (the time from admission to neurological symptoms) or intervention, blood pressure at the admission, and blood pressure before rebleeding/intervention. Moreover, the time interval from admission to rebleeding or intervention was recorded. Furthermore, Modified Fisher scale (mFS) and IA site were collected from the radiological data.

The morphology assessment was performed according to our previous studies (Liu et al., 2019; Chen S. et al., 2021; Yang et al., 2021). The reconstruction of vascular model and measurement of morphological parameters were conducted by two neurosurgeons (QL and YY, who were blind to clinical information and had worked as cerebral vascular neurosurgeons for more than 3 years). The Digital Imaging and Communications in Medicine data were introduced into Mimics 17.0 (Mimics Research 17.0, Materialize, Belgium) and then reconstructed for subsequent studies. The pathological protruding region was recognized as the IA sac and was separated from the parent artery for further analysis. Two neurosurgeons separated the IA sacs independently, the discrepancy was solved by consulting a senior neurosurgeon (HWH, who had worked in neurointervention for more than 15 years).

This study measured aneurysm size (S), diameter of dome (D), perpendicular height (H), diameter of parent artery, vessel angle (VA), aneurysm inclination angle (AA), volume, and surface area according to our previous study (Jiang et al., 2018a). The same neurosurgeons measured these morphological parameters independently, the discrepancy was solved by consulting a senior neurosurgeon (SW). The averages of measurements from each neurosurgeon were taken to be analyzed in depth. The morphological parameters involved here are also listed in Supplementary Table 1. Aspect ratio (AR), size ratio (SR), undulation index (UI), and non-sphericity index (NSI) were calculated according to previous study (Dhar et al., 2008). An irregular shape was defined as small bleb(s) or secondary aneurysm(s) protruding from the IA fundus or bi-/multi-lobular IA fundus.

### Computational Fluid Dynamics Simulating and Hemodynamic Assessment

The hemodynamic analysis protocol was referred to our previously conducted studies (Liu et al., 2019; Chen S. et al., 2021; Yang et al., 2021). For no saccular IAs sited in A3–A5 (anterior cerebral artery), M3–M5 (middle cerebral artery), P3–P4 (posterior cerebral artery), and vertebral artery in this study, we kept the vascular from internal carotid artery to M2 and A2 for IAs sited in anterior circulation and the vascular from basilar artery to P2 for IAs sited in posterior circulation. Meshing was performed using STAR-CCM (STAR-CCM + 12, Siemens, German), which automatically created 4 to 5 million unites of finite tetrahedral, prism elements and optimal boundary layers. The simulations were performed using STAR-CCM fluid workstation (STAR-CCM + 12, Siemens, Germany). The Navier-Stokers equation was employed as the solver in pulsatile blood. To conduct in-depth analyses, the pulsatile waveform of the internal carotid artery (waveform at cervical segmentation, for IAs sited in anterior circulation) and basilar artery (for IAs sited in posterior circulation) from a representative patient (Supplementary Figure 1) were adopted. The pulsatile waveform was obtained using Origin 2018b (OriginLab Corporation, Massachusetts, United States) and was exported to a comma-separated value file for further analysis. Blood was assumed as the incompressible Newtonian fluid. We set the blood as density ρ = 1056 kg/m^3^ and viscosity μ = 0.0035 Poise. Pulsatile curve was set as the velocity inlet boundary condition, and velocity boundary condition (the mass flow rate was obtained from a population-based study (Tegeler et al., 2013)) was set at the outlet. Under the residuals < 10^–5^, the results would be considered converged (Tian et al., 2016). A time step of 0.0001s was used. A cardiac cycle was divided into 800 steps (total 0.8 s per cycle). Four pulsatile cycles were simulated. The last cycle was yielded for subsequent studies.

Based on separated IA sacs, we extracted the time-averaged WSS and pressure over a cycle. The oscillatory shear index (OSI) and relative resident time (RRT) were calculated. In addition, the spatially average WSS, pressure, RRT and OSI over the aneurysm surface were obtained. Moreover, the hemodynamic parameters involved here are also listed in Supplementary Table 1. Specific to each model, WSS maximum (WSSM), WSS average (WSSA), WSS gradient (WSSG), and pressure average (PA), were obtained from the IA region, and parent pressure average, parent WSS average were determined according to the parent artery region. Low shear area was defined as the area with WSS < 10% of WSS of parent artery according to previous study (Xiang et al., 2011), and the percentage of low WSS area in IA dome (i.e., low shear area ratio, LSAR) was determined. The normalization of pressure and WSS was performed based on the hemodynamic status of parent artery. Furthermore, the normalized WSS average (NWSSA), normalized pressure average (NPA), and normalized WSS maximum (NWSSM) were calculated, respectively.

### Statistical Analysis

Measurement variables were compared using chi-square test or Fisher’s exact test. Continuous variables were compared using the independent samples’ t-test. PHASES score and ELAPSS score were calculated by complying with previous protocols (Greving et al., 2014; Backes et al., 2017). The parameters with significance in univariable analysis were inputted into Cox regression model to identify the independent risk factors. The result was expressed as hazard ratio (HR) and 95% confidence interval (CI). The performance of the nomograms to predict the rebleeding was measured with AUCs in receiver operating characteristic curve (ROC) analyses. An AUC > 0.7 was considered a clinical utility. The cutoff value was calculated using the Youden index. To further assess the predictive accuracy of nomograms, the risk, assessed by using nomogram models, was adopted to categorize patients as the high-risk group and the low-risk group at the risk as 50% (with the highest Youden index). The survival analysis was conducted by using Kaplan-Meier model. The statistical analyses were conducted by employing SPSS 24.0 (SPSS, Chicago, IL, United States), with two-sided *P* < 0.05 showing statistical significance.

Subsequently, nomograms to predict the rebleeding were formulated. All parameters of interest as described above fell to three categories, i.e., clinical, morphological and hemodynamic features (Supplementary Table 2). Based on results of the multivariate Cox regression analyses, this study developed two nomogram models incorporating factors independently associated with the primary endpoint, i.e., the clinical + morphological model (CM model) and clinical + morphological + hemodynamic model (CMH model). In addition, the calibration curves were plotted to assess the calibration of the nomograms. The nomograms were subjected to bootstrapping validation (1000 bootstrap resamples). Furthermore, the nomograms were developed with the package of “rms” in R version 3.6.2.

## Results

### Demographic, Clinical, Radiological, and Hemodynamic Difference in the Primary Cohort

**FIGURE 1:**
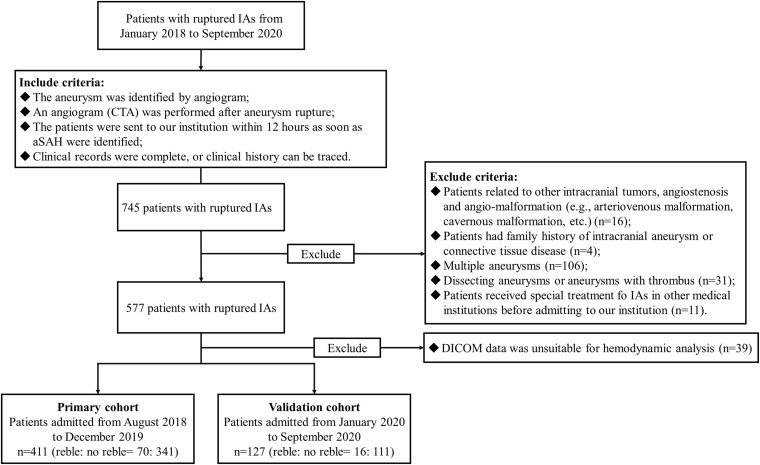
The study flow chart. In this study, 411 appropriate patients with ruptured IAs were reviewed. A total of 70 patients were identified as rebleeding after the admission. Patients enrolled from August 2018 to December 2019 formed the primary cohort (411 patients with 411 IAs), which was adopted to develop the predictive model; while patients enrolled from January 2020 to September 2020 were classified as the validation cohort (127 patients with 127 IAs). IAs, intracranial aneurysms; DICOM, Digital Imaging and Communications in Medicine data.

**FIGURE 2:**
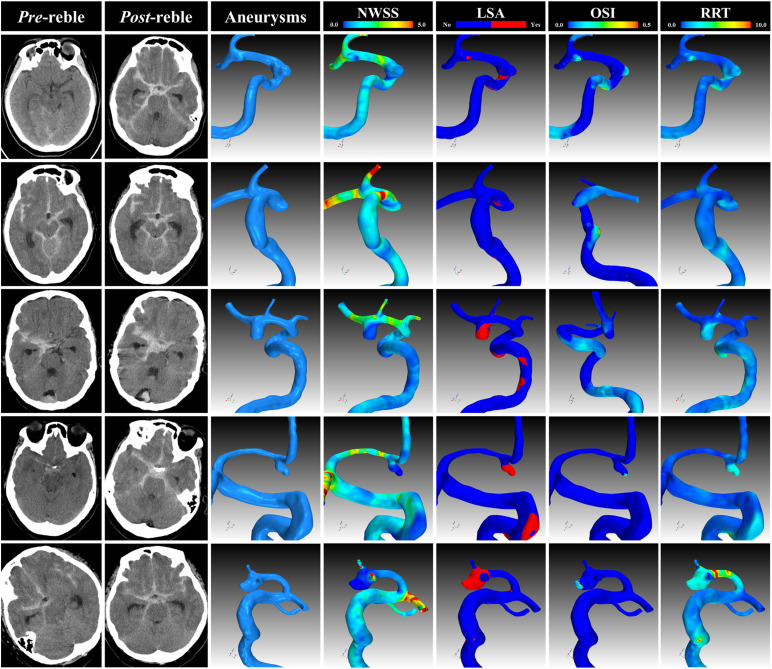
Representative cases. The CT, angiogram and hemodynamic analysis of several representative cases. CT, computational tomography; NWSS, normalized wall shear stress; LSA, low shear area; OSI, oscillatory shear index; RRT, relative resident time.

On the whole, 411 appropriate patients with ruptured IAs were reviewed (Figure 1). 70 patients were identified as rebleeding after the admission ranged in age from 49 to 68 years (mean: 58.4 ± 6.0 years). The rebleeding rate was 17.0%. The demographic and clinical information was given in Table 1. Of all patients in the primary cohort, the percentage of male patients was 39.2% (161/411). 34.1% (150/411) patients had hypertension. Several representative cases were presented in Figure 2.

**TABLE 1:** The demographic and baseline information of patients in the primary cohort.

Characteristics	With stable IAs *n* = 341	With rebleeding IAs *n* = 70	*P* value
Male, n (%)	129 (37.8%)	32 (45.7%)	0.219
Age, years, M ± SD	54.8 ± 10.4	54.0 ± 8.9	0.467
**Comorbidities, n (%)**			
Hypertension	111 (32.6%)	39 (11.4%)	<0.001^+^
Dyslipidemia	28 (8.2%)	10 (14.3%)	0.110
Diabetes mellitus	11 (3.2%)	3 (4.3%)	0.656
Coronary heart disease	8 (2.3%)	3 (4.3%)	0.360
Ischemic stroke	10 (2.9%)	3 (4.3%)	0.556
**Modified Fisher scale at admission, n (%)**			0.517
I–II	121 (35.5%)	22 (31.4%)	
III–IV	220 (64.5%)	48 (68.6%)	
**Hunt-Hess grade at admission, n (%)**			0.829
I–II	219 (64.2%)	44 (62.9%)	
III–V	122 (35.8%)	26 (37.1%)	
**Blood pressure**			
At admission, n (%)			0.578
<160/90 mmHg	114 (33.4%)	21 (30.0%)	
>160/90 mmHg	227 (66.6%)	49 (70.0%)	
Before rebleeding/surgery, n (%)			0.951
<140/80 mmHg	311 (91.2%)	64 (91.4%)	
>140/80 mmHg	30 (8.8%)	6 (8.6%)	
**Treatment, n (%)**			
Dead before surgical intervention	7 (2.1%)	14 (20.0%)	
Endovascular intervention	189 (55.4%)	36 (51.4%)	
Microsurgical clipping	145 (42.5%)	20 (28.6%)	
PHASES, m (IQR)	2 (0–4)	2 (1–5)	0.265
ELAPSS, m (IQR)	9 (5–14)	12.5 (7–15)	0.004^+^

*^+^The parameter was significant. IAs, intracranial aneurysms.*

**FIGURE 3:**
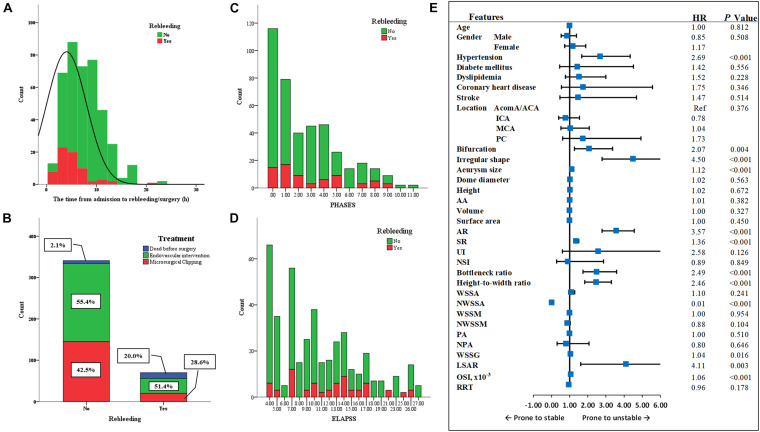
The factors related to the rebleeding after admission. **(A)** The average time from admission to the rebleeding was 4.4 ± 4.7 h. **(B)** 54.7% patients received endovascular intervention, and 40.2% patients received microsurgical clipping, whereas 5.1% died before the surgery, of which 66.7% patients underwent rebleeding after the admission. **(C,D)** The PHASES score and ELAPSS score were presented here. **(E)** The forest plot summarized the result of univariate Cox regression analysis. MCA, middle cerebral artery; ICA, internal carotid artery; AcomA, anterior communicating artery; ACA, anterior cerebral artery; PC, posterior circulation; AA, aneurysm inclination angle; AR, aspect ratio; SR, size ratio; UI, undulation index; NSI, non-sphericity index; WSSA, wall shear stress average; NWSSA, normalized wall shear stress average; WSSM, wall shear stress maximum; NWSSM, normalized wall shear stress maximum; PA, pressure average; NPA, normalized pressure average; WSSG, wall shear stress gradient; LSAR, low shear area ratio; OSI, oscillatory shear index; RRT, relative resident time; Ref, reference; HR, hazard ratio.

After standardly caring, the blood pressure of 91.2% (375/411) of patients was controlled in a reasonable range before rebleeding/intervention. The average time from admission to the rebleeding was 4.4 ± 4.7 h (Figure 3A). 54.7% (225/411) of patients received endovascular intervention, whereas 5.1% (21/411) died before the intervention, of which 66.7% (14/21) of patients underwent rebleeding after the admission (Figure 3B).

Table 2 lists the morphological and hemodynamic characteristics. Several characteristics, including the history of hypertension (*P* < 0.001), bifurcation (*P* = 0.005), irregular shape (*P* < 0.001), size (*P* < 0.001), AR (*P* < 0.001), SR (*P* < 0.001), bottleneck factor (*P* < 0.001), height-to-width ratio (*P* < 0.001), NWSSA (*P* < 0.001), WSSG (*P* = 0.008), LSAR (*P* = 0.010), OSI (*P* < 0.001), and RRT (*P* < 0.001), were significantly different between stable and rebleeding IAs.

**TABLE 2:** The morphological and hemodynamic features of IAs in the primary cohort.

	Stable IAs *n* = 341	Rebleeding IAs *n* = 70	*P* value
Location, n (%)			0.470
AcomA/ACA	52 (15.2%)	12 (17.1%)	
ICA	159 (46.6%)	27 (38.6%)	
MCA	117 (34.3%)	26 (37.1%)	
PC	13 (3.8%)	5 (7.1%)	
Bifurcation, n (%)	142 (41.6%)	42 (60.0%)	0.005^+^
Irregular shape, n (%)	58 (17.0%)	38 (54.3%)	<0.001^+^
IAsize, mm, M ± SD	5.6 ± 2.9	7.0 ± 2.7	<0.001^+^
Dome diameter, mm, M ± SD	4.9 ± 3.5	5.0 ± 3.1	0.248
Height, mm, M ± SD	4.5 ± 2.4	4.7 ± 1.9	0.057
AA,°, M ± SD	88.8 ± 19.0	91.3 ± 23.2	0.859
Volume, mm^3^, m (IQR)	38.4 (19.7–69.4)	40.1 (22.3–90.5)	0.958
Surface area, mm^2^, m (IQR)	54.8 (33.2–132.1)	50.2 (33.4–180.9)	0.949
AR, M ± SD	1.2 ± 0.4	2.1 ± 0.8	<0.001^+^
SR, M ± SD	2.1 ± 1.4	3.1 ± 2.2	<0.001^+^
UI, M ± SD	0.3 ± 0.2	0.4 ± 0.3	0.541
NSI, M ± SD	0.3 ± 0.2	0.3 ± 0.2	0.732
Bottleneck factor, M ± SD	1.2 ± 0.4	1.5 ± 0.5	<0.001^+^
Height-to-width ratio, M ± SD	1.5 ± 0.5	2.0 ± 0.9	<0.001^+^
WSSA, Pa, m (IQR)	2.2 (1.2–3.7)	2.7 (1.3–4.1)	0.093
NWSSA, m (IQR)	0.42 (0.26–0.63)	0.22 (0.18–0.28)	<0.001^+^
WSSM, Pa, m (IQR)	6.4 (4.0–9.9)	6.7 (3.3–9.8)	0.580
NWSSM, m (IQR)	1.4 (0.68–2.8)	1.0 (0.57–2.6)	0.227
PA, kPa, m (IQR)	2.3 (1.6–2.8)	2.1 (1.3–2.5)	0.173
NPA, m (IQR)	0.62 (0.44–0.80)	0.57 (0.44–0.77)	0.184
WSSG, m (IQR)	23.0 (16.5–29.1)	16.5 (14.2–25.8)	0.008^+^
LSAR, m (IQR)	0.28 (0.15–0.45)	0.38 (0.23–0.54)	0.010^+^
OSI, x10^–2^, m (IQR)	0.58 (0.20–0.97)	0.70 (0.43–1.73)	<0.001^+^
RRT, m (IQR)	5.79 (3.74–8.22)	4.57 (3.02–7.80)	0.059

*^+^The parameter with significant.*

*IAs, intracranial aneurysms; MCA, middle cerebral artery; ICA, internal carotid artery; AcomA, anterior communicating artery; ACA, anterior cerebral artery; PC, posterior circulation; AA, aneurysm inclination angle; AR, aspect ratio; SR, size ratio; UI, undulation index; NSI, non-sphericity index; WSSA, wall shear stress average; NWSSA, normalized wall shear stress average; WSSM, wall shear stress maximum; NWSSM, normalized wall shear stress maximum; PA, pressure average; NPA, normalized pressure average; WSSG, wall shear stress gradient; LSAR, low shear area ratio; OSI, oscillatory shear index; RRT, relative resident time.*

The ELAPSS score was significantly higher in rebleeding IAs compared with stable IAs [12.5 (7–15) vs. 9 (5–14), *P* = 0.004]; however, the PHASES score was not significant between rebleeding and stable IAs (*P* = 0.265). Figures 3C,D present the distributions of rebleeding cases in PHASES score and ELAPSS score.

### Risk Factors of Rebleeding After the Admission

The significant parameters in univariate analysis were inputted into univariate Cox regression model. The results are summarized as a forest plot (Figure 3E). Hypertension (*P* < 0.001), bifurcation (*P* = 0.004), irregular shape (*P* < 0.001), size (*P* < 0.001), AR (*P* < 0.001), SR (*P* < 0.001), bottleneck ratio (*P* < 0.001), height-to-width ratio (*P* < 0.001), NWSSA (*P* < 0.001), WSSG (*P* = 0.016), LSAR (*P* = 0.003), and OSI (*P* < 0.001) were identified as the risk factors of rebleeding after the admission.

These parameters were then inputted into a multivariate Cox regression model. The result was summarized in Table 3. The parameters were demonstrated as independent risk factors for the rebleeding after the admission, including hypertension (HR = 2.54; 95% CI, 1.02–6.31, *P* = 0.044), bifurcation (HR = 1.95; 95% CI, 1.12–3.07, *P* = 0.013), irregular shape (HR = 4.22; 95% CI, 1.68–10.62, *P* = 0.002), AR (HR = 12.91; 95% CI, 4.74–35.13, *P* < 0.001), and NWSSA (HR = 0.16; 95% CI, 0.01–0.28, *P* = 0.002), as well as OSI (HR = 1.14; 95% CI, 1.06–1.23, *P* < 0.001).

**TABLE 3:** Multivariate Cox analysis for rebleeding before surgery in the primary cohort.

Characteristics	HR	95% CI	*P* value
Hypertension (Yes vs. No)	2.54	(1.02–6.31)	0.044^+^
Bifurcation (Yes vs. No)	1.95	(1.12–3.07)	0.013^+^
Irregular shape (Yes vs. No)	4.22	(1.68–10.62)	0.002^+^
Aneurysm size	1.07	(0.90–1.28)	0.450
AR	12.91	(4.74–35.13)	<0.001^+^
SR	0.93	(0.65–1.34)	0.659
Bottleneck ratio	0.93	(0.35–2.42)	0.704
Height-to-width ratio	3.52	(0.73–16.97)	0.496
NWSSA	0.16	(0.01–0.28)	0.002^+^
WSSG	1.09	(0.93–1.26)	0.153
LSAR	0.15	(0.01–2.87)	0.226
OSI	1.14	(1.06–1.23)	<0.001^+^

*^+^The independent risk factor associated with rebleeding.*

*AR, aspect ratio; SR, size ratio; NWSSA, normalized wall shear stress average; WSSG, wall shear stress gradient; LSAR, low shear area ratio; OSI, oscillatory shear index.*

### Nomogram Models to Predict the Rebleeding After the Admission

**FIGURE 4:**
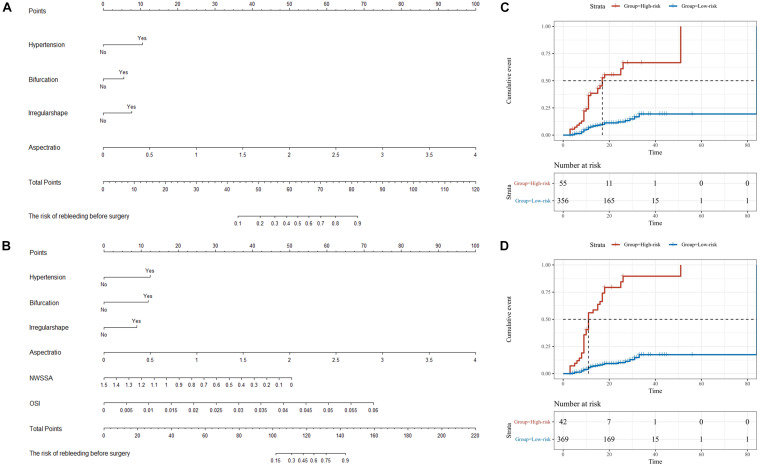
The nomograms for the risk of rebleeding after admission. **(A,B)** Two nomograms, i.e., clinical + morphological model (CM model) and clinical + morphological + hemodynamic model (CMH model), were presented here. **(C,D)** With the risk at 50%, all patients were categorized as the high-risk group and the low-risk group. The survival curves showed that the RIAs in the high-risk group might rebleed in a short period after admission. NWSSA, normalized wall shear stress average; OSI, oscillatory shear index.

**FIGURE 5:**
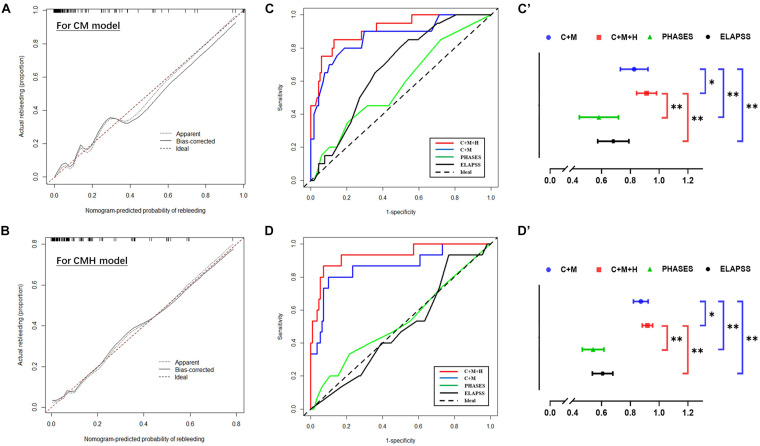
The predictive accuracy of models for rebleeding. **(A,B)** The calibration plots display a substantial agreement between the prediction by each nomogram and the actual observation, in the risk of rebleeding before the surgery. **(C,C’,D,D’)** The ROC analyses based on the primary cohort and validation cohort showed a good predicting accuracy of two nomogram models, whereas a poor predicting accuracy of PHASES and ELAPSS score was found. ^∗^*P* < 0.05; ^∗∗^*P* < 0.01.

Based on the result of multivariate Cox regression analysis and parameter category, two nomograms, i.e., clinical + morphological model (CM model, Figure 4A) and clinical + morphological + hemodynamic model (CMH model, Figure 4B) were built. With the risk as 50%, all patients were categorized as the high-risk group and the low-risk group. The survival curves are presented as Figures 4C,D. Here, the patients in the high-risk group, recognized by the CM model and CMH model respectively, had higher risk of rebleeding after the admission (both *P* < 0.001). The calibration plots display a substantial agreement between the prediction by each nomogram and the actual observation, in the risk of rebleeding before the intervention (Figures 5A,B). The ROC analyses based on the primary cohort showed a good predicting accuracy of two nomogram models (AUC = 0.83 and 0.92, respectively), whereas a poor predicting accuracy of PHASES and ELAPSS score was found (AUC = 0.54 and 0.61, respectively). The CMH model had a higher predicting accuracy compared with the CM model (*P* < 0.05). The results of ROC analyses are summarized as Figures 5C,C’ and given in Table 4.

**TABLE 4:** The predicting value of each model.

	Primary cohort			Validation cohort		
	AUC	95%CI	*P* value	AUC	95%CI	*P* value
**Nomogram models**						
CM model^*a*^	0.83	(0.78–0.90)	<0.001	0.86	(0.75–0.98)	<0.001
CMH model^*b*^	0.92	(0.88–0.95)	<0.001	0.93	(0.86–1.00)	<0.001
**Clinical scores**						
PHASES	0.54	(0.47–0.62)	0.273	0.53	(0.36–0.70)	0.712
ELAPSS	0.61	(0.54–0.68)	0.004	0.51	(0.36–0.65)	0.917

*^*a*^CM model, clinical and morphological model.*

*^*b*^CMH model, clinical, morphological and hemodynamic model.*

*AUC, area under the curve; CI, confidence interval.*

### Validation of Predicting Accuracy of Nomogram Models to the Rebleeding After the Admission

The information of the validation cohort was given in Table 5. No significant difference was identified between the primary cohort and the validation cohort (Supplementary Table 3). As indicated from the ROC analyses (Figures 5D,D’), the CMH model exhibited the highest predicting accuracy (AUC = 0.93), followed by CM model (AUC = 0.86); however, the PHASES and ELAPSS score performed poorly (AUC = 0.53 and 0.51, respectively) in predicting the rebleeding after the admission. The result of ROC analyses based on the validation cohort is also listed in Table 4.

**TABLE 5:** The information of patients and IAs in the validation cohort.

Characteristics	Stable IAs *n* = 111	Rebleeding IAs *n* = 16	*P* value
Age, years, M ± SD	54.6 ± 10.5	53.4 ± 5.8	0.588
Male, n (%)	43 (38.7%)	8 (50.0%)	0.392
**Comorbidities, n (%)**			
Hypertension	40 (36.0%)	10 (62.5%)	0.044^+^
Dyslipidemia	9 (8.1%)	3 (18.8%)	0.175
Diabetes mellitus	5 (4.5%)	1 (6.3%)	0.759
Coronary heart disease	2 (1.8%)	0 (0.0%)	0.590
Ischemic stroke	3 (2.7%)	1 (6.3%)	0.449
History of aSAH	16 (14.4%)	2 (12.5%)	0.837
Modified Fisher scale at admission, n (%)			0.742
I–II	37 (33.3%)	6 (37.5%)	
III–IV	74 (66.7%)	10 (62.5%)	
Hunt-Hess grade at admission, n (%)			0.106
I–II	67 (60.4%)	13 (81.2%)	
III–V	44 (39.6%)	3 (18.8%)	
**Blood pressure**			
At admission, n (%)			0.117
<160/90 mmHg	35 (31.5%)	2 (12.5%)	
>160/90 mmHg	76 (68.5%)	14 (87.5%)	
Before rebleeding/surgery, n (%)			0.574
<140/80 mmHg	99 (89.2%)	15 (93.8%)	
>140/80 mmHg	12 (9.8%)	1 (6.2%)	
Location, n (%)			0.244
AcomA/ACA	16 (14.4%)	5 (31.3%)	
ICA	53 (47.7%)	6 (37.5%)	
MCA	40 (36.1%)	5 (31.3%)	
PC	2 (1.8%)	0 (0.0%)	
Bifurcation, n (%)	42 (37.8%)	13 (81.3%)	0.001^+^
Irregular shape, n (%)	17 (15.3%)	9 (56.3%)	<0.001^+^
IAsize, mm, M ± SD	4.7 (3.8–7.1)	6.4 (4.5–7.5)	0.130
Dome diameter, mm, M ± SD	3.9 (2.9–5.6)	4.3 (3.2–6.0)	0.472
Height, mm, M ± SD	3.8 (3.0–5.7)	3.9 (3.5–5.4)	0.520
AA,°, M ± SD	86.8 (79.9–98.3)	85.6 (81.3–103.3)	0.757
Volume, mm^3^, m (IQR)	41.6 (19.7–121.9)	40.3 (29.3–75.5)	0.870
Surface area, mm^2^, m (IQR)	60.7 (34.6–144.84)	52.2 (46.6–161.0)	0.951
AR, m (IQR)	1.2 (0.9–1.5)	1.9 (1.3–2.3)	<0.001^+^
SR, m (IQR)	1.6 (1.2–2.6)	2.3 (1.3–5.0)	1.116
UI, m (IQR)	0.26 (0.15–0.42)	0.19 (0.12–0.49)	0.525
NSI, m (IQR)	0.29 (0.06–0.44)	0.21 (0.07–0.43)	0.525
Bottleneck factor, m (IQR)	1.1 (1.0–1.3)	1.3 (1.1–1.7)	0.064
HWR, m (IQR)	1.4 (1.2–1.7)	1.9 (1.4–2.5)	0.024^+^
WSSA, Pa, m (IQR)	2.3 (1.4–4.0)	2.2 (1.0–3.8)	0.773
NWSSA, m (IQR)	0.42 (0.26–0.61)	0.22 (0.18–0.27)	0.001^+^
WSSM, Pa, m (IQR)	6.7 (3.9–11.3)	6.0 (2.9–10.0)	0.606
NWSSM, m (IQR)	1.31 (0.68–2.38)	1.25 (0.65–2.45)	0.922
PA, Pa, m (IQR)	2319.3 (1737.6–2910.7)	1912.9 (1270.8–2217.9)	0.008^+^
NPA, m (IQR)	0.60 (0.42–0.76)	0.64 (0.44–0.77)	0.719
WSSG, m (IQR)	6.9 (5.4–10.7)	7.9 (6.3–13.0)	0.1123
LSAR, m (IQR)	0.27 (0.16–0.44)	0.41 (0.26–0.55)	0.072
OSI, x10^–2^, m (IQR)	0.60 (0.17–0.91)	0.84 (0.51–1.64)	0.009^+^
RRT, m (IQR)	5.3 (3.6–7.2)	4.5 (3.0–5.9)	0.286

*^+^The parameter was significant.*

*IAs, intracranial aneurysms; MCA, middle cerebral artery; ICA, internal carotid artery; AcomA, anterior communicating artery; ACA, anterior cerebral artery; PC, posterior circulation; AA, aneurysm inclination angle; AR, aspect ratio; SR, size ratio; UI, undulation index; NSI, non-sphericity index; WSSA, wall shear stress average; NWSSA, normalized wall shear stress average; WSSM, wall shear stress maximum; NWSSM, normalized wall shear stress maximum; PA, pressure average; NPA, normalized pressure average; WSSG, wall shear stress gradient; LSAR, low shear area ratio; OSI, oscillatory shear index; RRT, relative resident time.*

## Discussion

Rebleeding refers to a main cause of morbidity for aSAH patients. Existing study reported that the morphology and hemodynamics of IAs would change after aSAH, which makes IAs prone to be stable (Skodvin et al., 2017). However, some RIAs may not reach a stable condition and had a high risk of rebleeding. In this study, we confirmed the predictive value of hemodynamic parameters for rebleeding after the admission and build a predicting model to discriminate the RIAs at high risk of rebleeding.

This study demonstrated the relationship between the history of hypertension and rebleeding after the admission. Systematic artery hypertension was recognized as the major cause of cardiovascular disease. Previous cohort and animal studies confirmed that hypertension could increase the risk of IA natural rupture (Lindgren et al., 2014; Tada et al., 2014); thus, the hypertension was considered in subsequent predicting models (Greving et al., 2014; Backes et al., 2017). Here, the hypertension was also found as the independent risk factor for the rebleeding after the admission, demonstrating that the risk of rebleeding of RIA patients with hypertension was approximately 2.7 times that of patients without hypertension. Though the blood pressure was well under control after the admission, the systematic artery hypertension had caused damage to vessels throughout the body before the IAs rupture. Accordingly, this study considered that the risk of rebleeding was higher in patients with hypertension as compared to patients without hypertension.

In this study, the RIAs sited in bifurcation, with irregular shape, and larger AR were found with a high risk of rebleeding after the admission. The IAs sited in bifurcation were more possible to suffer from the impact of blood flow; the dynamic change from direct impact area to surrounding area could cause physical injury to the endothelia of vessels, and thus to the aneurysm wall (Metaxa et al., 2010). In addition to the bifurcation site, irregular shape is also a sign of high risk of rupture. Irregular shape generally suggested a more significantly fragile area in IAs, which could present as bled or second aneurysm protruding from the primary IAs, as compared with the surrounding area in the aneurysm dome. As revealed from existing studies, the bled or second aneurysm in an irregular aneurysm was generally thin and blood-blister like, and the rupture areas were commonly associated with these bled or second aneurysms (Kawaguchi et al., 2012; Jiang et al., 2020). Notably, the hemodynamic condition of the bled aneurysm is generally low WSS and high OSI (Kawaguchi et al., 2012). Thus, it is easy to understand that the RIAs sited in bifurcation with an irregular shape has a higher risk of rebleeding after the admission. The IAs with large AR generally have large size and relatively narrow neck, often with unstable hemodynamic condition and severe damage in the aneurysm wall (Qiu et al., 2017); therefore, this parameter was confirmed as a predictor for IA natural rupture (Backes et al., 2017). Our preliminary study also reported that the AR was a predictor for rebleeding of RIAs. This study demonstrated that the risk of rebleeding increased by 12.9 per 1 of AR, suggesting that AR is a vital parameter to identify the RIAs at high risk of rebleeding after the admission.

This study also confirmed the role of hemodynamic parameters, mainly the WSSA and OSI, in predicting the risk of rebleeding after the admission. The hemodynamic condition could induce inflammation filtration and vessel remodeling in the vascular wall (Albarran-Juarez et al., 2018; Lu et al., 2019; Zhang et al., 2020), which would cause atherosclerosis, IA growth/rupture, etc. Recent studies reported that oscillator flow could induce the inflammation in the vascular wall and inhibit the expression of vascular protective factors (Albarran-Juarez et al., 2018) (e.g., endothelial nitric oxide synthase). Moreover, the low WSS could activate the inflammatory pathway (Chen J. et al., 2021) (e.g., ROS pathway, pyroptosis pathway and nuclear factor kappa B pathway) to induce inflammation, which could promote the damage of vessels. This study demonstrated that the RIAs with low WSSA and high OSI had a high risk of rebleeding after the admission. Notably, as suggested by existing pathological research, the inflammation in the aneurysm wall is the main cause of IA rupture and growth (Frösen et al., 2004, 2012; Jiang et al., 2018b; Tulamo et al., 2018). According to Frosen et al., the pathological characteristics of the aneurysm wall fell to four levels by largely complying with the inflammation; the severer the level, the higher the risk of aneurysm rupture will be (Frösen et al., 2004). Since WSS and OSI could induce the inflammation in the vascular wall, the low WSS and the high OSI in RIAs suggested that the damage for the aneurysm wall was continuous after initial hemorrhage; besides, for rebleeding aneurysms, the bleeding stopped before IA reaching a stable status. The mentioned result also demonstrated the clinical utility of computational fluid dynamics.

Based on the independent risk factors, two nomogram models were set to identify the RIAs at high risk of rebleeding. The CM model included the clinical and morphological characteristics (e.g., hypertension, bifurcation site, irregular shape, and AR). The hemodynamic characteristics were further integrated with the other characteristics to build the CMH model. The predicting value of the CM model and CMH model was confirmed as good for clinical utility. Using the risk as 50% assessed by each nomogram model, we confirmed that the rupture risk was higher and interval from initial hemorrhage to rebleeding was shorter in the high-risk group as compared with the low-risk group. As indicated from the further comparison, these two nomogram models exhibited higher predicting accuracy as compared with PHASES and ELAPSS models. Interestingly, the CMH model had higher predicting accuracy as compared with the CM model. For this phenomenon, this study considered that the stability of IAs mainly involved two aspects, i.e., internal hemodynamic condition and pathological characteristics of the aneurysm wall. Taking multidimensional risk factors into consideration could help to comprehensively understand the stability of RIAs, demonstrating that the CMH model would have a higher predicting accuracy as compared with the CM model. However, the CM model could be more easily handled in clinical work as compared with the CMH model, especially in emergency conditions. Though the hemodynamic analysis is limited in clinical work for its technical barrier and time-consumption now, the tool, i.e., aView, has been reported in previous study (Xiang et al., 2017); therefore, the clinical practical hemodynamic analysis tool would arise to assist in quickly identifying the hemodynamic characteristics of RIAs in the future.

In the developing nations, because of large populations but limited medical resources (Bian et al., 2012), the sequence of treatment is essential to make a treatment strategy for IAs. This fact reveals that a patient has to wait a long time for appropriate treatment immediately to get medical intervention after IA rupture. Though the bleeding stopped in some RIAs, RIAs may not reach a real stable condition. This study built two models with good accuracy to identify the high-risk RIAs. To avoid rebleeding, an immediate surgical intervention was recommended for the RIAs at high risk of rebleeding, and a priority should be given to IAs with higher scores.

There are several limitations here. First, the inlet boundary condition was from a representative patient, which can affect the result of computational fluid dynamics since this method is sensitive to velocity and waveform (Xiang et al., 2014). However, this study used normalized parameters that can reduce the effects exerted by this problem. Second, the morphology can significantly impact the hemodynamics. Due to the change of morphology and hemodynamics after indiscoverable IA rupture (Skodvin et al., 2017) and the effect of hemorrhage on the quality of radiological images, our conclusion may be limited. Third, this study was a single center and retrospective study, which may limit our conclusion. Fourth, this study only considered the utility of PHASES and ELAPSS score in predicting the risk of rebleeding. There were several other models which could help in discriminating the high-risk IAs, e.g., Detmer’s model (Detmer et al., 2018). However, the PHASES and ELAPSS score were representative models to discriminate the high-risk IAs; thus, we compared the predictive accuracy between our nomograms and these models. Indications remain the focus in IA treatment, and our models have their clinical utility to help clinical work identify the optimal sequence of treatment, though some limitations remain.

## Conclusion

Hemodynamic parameters could serve as the predictors for rebleeding after admission. Two nomogram models were presented, i.e., CMH nomogram and CM nomogram, helping to identify the RIAs at high risk of rebleeding. For RIAs at high risk of rebleeding, intervention should be prioritized, and medical treatment is not recommended after rupture.

## Data Availability Statement

The original contributions presented in the study are included in the article/Supplementary Material, further inquiries can be directed to the corresponding authors.

## Ethics Statement

This study was approved by the Institutional Review Board of Beijing Tiantan Hospital. Written informed consents were obtained and the privacy of patients was effectively protected.

## Author Contributions

QL and PJ: conception and design. QL, YY, JY, PJ, ML, SY, and NW: acquisition of data. QL and YY: analysis and interpretation of data. QL: drafting the article. PJ and SW: critically revising the article. SW: approving the final version of the manuscript on behalf of other authors and study supervision. All authors: reviewing the submitted version of manuscript.

## Conflict of Interest

The authors declare that the research was conducted in the absence of any commercial or financial relationships that could be construed as a potential conflict of interest. The reviewer XY declared a shared affiliation, with no collaboration with the authors to the handling editor at the time of the review.

## Publisher’s Note

All claims expressed in this article are solely those of the authors and do not necessarily represent those of their affiliated organizations, or those of the publisher, the editors and the reviewers. Any product that may be evaluated in this article, or claim that may be made by its manufacturer, is not guaranteed or endorsed by the publisher.

## Abbreviations

IA: intracranial aneurysm
RIA: ruptured intracranial aneurysm
aSAH: aneurysmal subarachnoid hemorrhage
CT: computational tomography
mFS: modified Fisher scale
AR: aspect ratio
SR: size ratio
NSI: non-sphericity index
UI: undulation index
WSS: wall shear stress
WSSA: wall shear stress average
WSSM: wall shear stress maximum
PA: pressure average
WSSG: wall shear stress gradient
NWSSA: normalized wall shear stress average
NWSSM: normalized wall shear stress maximum
NPA: normalized pressure average
LSAR: low shear area rate
RRT: relative resident time
OSI: oscillatory shear index
VA: vessel angle
AA: aneurysm angle.
