# Supplementary material for: Rebleeding of Ruptured Intracranial Aneurysm After Admission: A Multidimensional Nomogram Model to Risk Assessment
Source: Front Aging Neurosci. 2021 Sep 1;13:692615. doi: 10.3389/fnagi.2021.692615 (PMC8440913; doi:10.3389/fnagi.2021.692615)
Supplement: Supplementary file 1 [file Data_Sheet_1.docx]

**Supplementary materials.**

**To Rebleeding of ruptured intracranial aneurysm after admission: a multidimensional nomogram model to risk assessment**

**Supplementary Table 1. The calculation formulas involved in this study**

|  | Abbreviation | Formula |
| --- | --- | --- |
| Aspect ratio* | AR | $\frac{IA size (S)}{Neck diameter}$ |
| Size ratio* | SR | $\frac{IA height (H)}{Diameter of parent artery}$ |
| Height-to-width ratio | - | $\frac{IA height (H)}{Neck diameter}$ |
| Undulation index* | UI | $1-\frac{IA volume}{Convex hull volume}$ |
| Nonsphericity index* | NSI | $1-{(18\pi)}^{1/3}\frac{{IA volume}^{2/3}}{IA surface area}$ |
| Oscillatory shear index | OSI | $\frac{1}{2}\left( 1-\frac{\left\vert\int_{0}^{T} {wss}_{i}dt \right\vert}{\int_{0}^{T} \left\vert{wss}_{i} \right\vert dt} \right)$ |
| Relative resident time | RRT | $\frac{1}{(1-2\times OSI)\times WSS}=\frac{1}{\frac{1}{T}\left\vert\int_{0}^{T} {wss}_{i}dt \right\vert}$ |
| Normalized wall shear stress average | NWSSA | $\frac{Spatial WSS average}{Spatial WSS average of parent artery}$ |
| Normalized wall shear stress maximum | NWSSM | $\frac{WSS maximum}{WSS maximum of parent artery}$ |
| Normalized pressure average | NPA | $\frac{Spatial pressure average}{Spatial pressure average of parent artery}$ |
| Low shear area rate | LSAR | $\frac{{Area}_{WSS<10\% of WSSA of parent artery}}{{Area}_{dome}}$ |

*, More details of the calculation methods could be found in Dhar et al’ s study (Dhar S, Tremmel M, Mocco J, Kim M, Yamamoto J, Siddiqui AH, et al. Morphology parameters for intracranial aneurysm rupture risk assessment. Neurosurgery. 2008; 63:185-196).

**Abbreviation:** IA, intracranial aneurysm; WSS, wall shear stress average.

**Supplementary Table 2. Summary of all parameters of interest**

| Categories of the parameters | Parameters |
| --- | --- |
| Clinical | Age |
|  | Male |
|  | Hypertension |
|  | Dyslipidemia |
|  | Diabetes mellitus |
|  | Coronary heart disease |
|  | Ischemic stroke |
| Morphological | Location |
|  | Bifurcation |
|  | Irregular shape |
|  | IA size |
|  | Dome diameter |
|  | Height |
|  | Vessel angle |
|  | Aneurysm inclination angle |
|  | Aneurysm volume |
|  | Surface area |
|  | Aspect ratio |
|  | Size ratio |
|  | Undulation index |
|  | Nonsphericity index |
|  | Bottleneck factor |
|  | Height-to-width ratio |
| Hemodynamic | WSSA |
|  | NWSSA |
|  | WSSM |
|  | NWSSM |
|  | PA |
|  | NPA |
|  | WSSG |
|  | LSAR |
|  | OSI |
|  | RRT |

**Abbreviation:** IA, intracranial aneurysm; WSSA, wall shear stress average; NWSSA, normalized wall shear stress average; WSSM, wall shear stress maximum; NWSSM, normalized wall shear stress maximum; PA, pressure average; NPA, normalized pressure average; WSSG, wall shear stress gradient; LSAR, low shear area ratio; OSI, oscillatory shear index

**Supplementary Table 3. The comparison of clinical, morphological and hemodynamic features between the primary cohort and validation cohort**

| Characteristics | Primary cohort  n=411 | | Validation cohort  n=127 | P value | |
| --- | --- | --- | --- | --- | --- |
| Age, years, m±SD | 54.6±10.1 | | 54.5±10.0 | 0.826 | |
| Male, n (%) | 161 (39.2%) | | 51 (40.2%) | 0.843 | |
| Comorbidities, n (%) |  | |  |  | |
| Hypertension | 150 (36.5%) | 50 (39.4%) | | 0.588 |  |
| Dyslipidemia | 38 (9.2%) | 12 (9.4%) | | 0.945 |  |
| Diabetes mellitus | 14 (3.4%) | 6 (4.7%) | | 0.493 |  |
| Coronary heart disease | 11 (2.7%) | 2 (1.6%) | | 0.480 |  |
| Ischemic stroke | 13 (3.2%) | 4 (3.1%) | | 0.994 |  |
| History of aSAH | 58 (14.1%) | | 18 (14.2%) | 0.986 | |
| Modified Fisher scale at admission, n (%) |  | |  | 0.846 | |
| I-II | 143 (34.8%) | | 43 (33.9%) |  | |
| III-IV | 268 (65.2%) | | 84 (66.1%) |  | |
| Hunt-Hess grade at admission, n (%) |  | |  | 0.838 | |
| I-II | 263 (64.0%) | | 80 (63.0%) |  | |
| III-V | 148 (36.0%) | | 47 (37.0%) |  | |
| Blood pressure |  | |  |  | |
| At admission, n (%) |  | |  | 0.433 | |
| <160/90mmHg | 135 (32.8%) | | 37 (29.1%) |  | |
| >160/90mmHg | 276 (67.2%) | | 90 (70.9%) |  | |
| Before rebleeding/surgery, n (%) |  | |  | 0.613 | |
| <140/80mmHg | 375 (91.2%) | | 114 (89.8%) |  | |
| >140/80mmHg | 36 (8.8%) | | 13 (10.2%) |  | |
| Location, n (%) |  | |  | 0.530 | |
| AcomA/ACA | 64 (15.6%) | | 21 (16.5%) |  | |
| ICA | 186 (45.3%) | | 59 (46.5%) |  | |
| MCA | 143 (34.8%) | | 45 (35.4%) |  | |
| PC | 18 (4.4%) | | 2 (1.6%) |  | |
| Lateralization, n (%) |  | |  | 0.897 | |
| Left | 205 (49.9%) | | 65 (51.2%) |  | |
| Right | 151 (36.7%) | | 47 (37.0%) |  | |
| Middle | 55 (13.4%) | | 15 (11.8%) |  | |
| Bifurcation, n (%) | 227 (55.2%) | | 72 (56.7%) | 0.772 | |
| Irregular shape, n (%) | 96 (23.4%) | | 26 (20.5%) | 0.498 | |
| S, mm, m (IQR) | 5.2 (3.9-7.1) | | 4.8 (3.8-7.0) | 0.916 | |
| D, mm, m (IQR) | 3.8 (2.9-5.7) | | 4.1 (3.0-5.7) | 0.431 | |
| H, mm, m (IQR) | 3.8 (3.1-5.6) | | 3.8 (3.1-5.4) | 0.841 | |
| AA, °, m (IQR) | 15.0 (11.3-24.6) | | 14.5 (12.0-31.7) | 0.767 | |
| Volume, mm^3^, m (IQR) | 39.6 (19.8-75.3) | | 40.5 (19.8-97.7) | 0.482 | |
| Surface area, mm^2^, m (IQR) | 54.2 (33.2-133.5) | | 58.0 (37.1-144.8) | 0.349 | |
| AR, m (IQR) | 1.2 (0.9-1.7) | | 1.2 (1.0-1.5) | 0.608 | |
| SR, m (IQR) | 1.7 (1.2-2.7) | | 1.6 (1.2-2.9) | 0.715 | |
| UI, m (IQR) | 0.3 (0.2-0.5) | | 0.3 (0.2-0.4) | 0.526 | |
| NSI, m (IQR) | 0.2 (0.1-0.4) | | 0.3 (0.1-0.4) | 0.307 | |
| Bottleneck factor, m (IQR) | 1.2 (1.0-1.4) | | 1.1 (1.0-1.4) | 0.684 | |
| HWR, m (IQR) | 1.4 (1.2-1.9) | | 1.4 (1.2-1.8) | 0.792 | |
| WSSA, Pa, m (IQR) | 2.3 (1.2-3.7) | | 2.3 (1.3-4.0) | 0.680 | |
| NWSSA, m (IQR) | 0.4 (0.2-0.6) | | 0.4 (0.3-0.6) | 0.664 | |
| WSSM, Pa, m (IQR) | 6.3 (3.6-10.0) | | 6.7 (3.3-10.9) | 0.561 | |
| NWSSM, m (IQR) | 1.4 (0.7-2.6) | | 1.3 (0.7-2.4) | 0.506 | |
| PA, kPa, m (IQR) | 2.1 (1.6-2.8) | | 2.3 (1.7-2.8) | 0.428 | |
| NPA, m (IQR) | 0.61 (0.44-0.77) | | 0.60 (0.42-0.76) | 0.530 | |
| WSSG, m (IQR) | 7.1 (5.4-10.7) | | 7.3 (4.6-9.7) | 0.941 | |
| LSAR, m (IQR) | 0.3 (0.2-0.5) | | 0.3 (0.2-0.5) | 0.934 | |
| OSI, x10^-2^, m (IQR) | 0.6 (0.2-1.0) | | 0.5 (0.2-0.9) | 0.771 | |
| RRT, m (IQR) | 5.4 (3.7-8.0) | | 5.2 (3.6-7.1) | 0.279 | |

**Abbreviation:** MCA, middle cerebral artery; ICA, internal carotid artery; AcomA, anterior communicating artery; ACA, anterior cerebral artery; PC, posterior circulation; S, aneurysm size; D, the diameter of body; H, the perpendicular height; VA, vessel angle; AA, aneurysm inclination angle; AR, aspect ratio; SR, size ratio; UI, undulation index; NSI, nonsphericity index; WSSA, wall shear stress average; NWSSA, normalized wall shear stress average; WSSM, wall shear stress maximum; NWSSM, normalized wall shear stress maximum; PA, pressure average; NPA, normalized pressure average; WSSG, wall shear stress gradient; LSAR, low shear area ratio; OSI, oscillatory shear index; RRT, relative resident time**.**

**Supplementary Figure.**


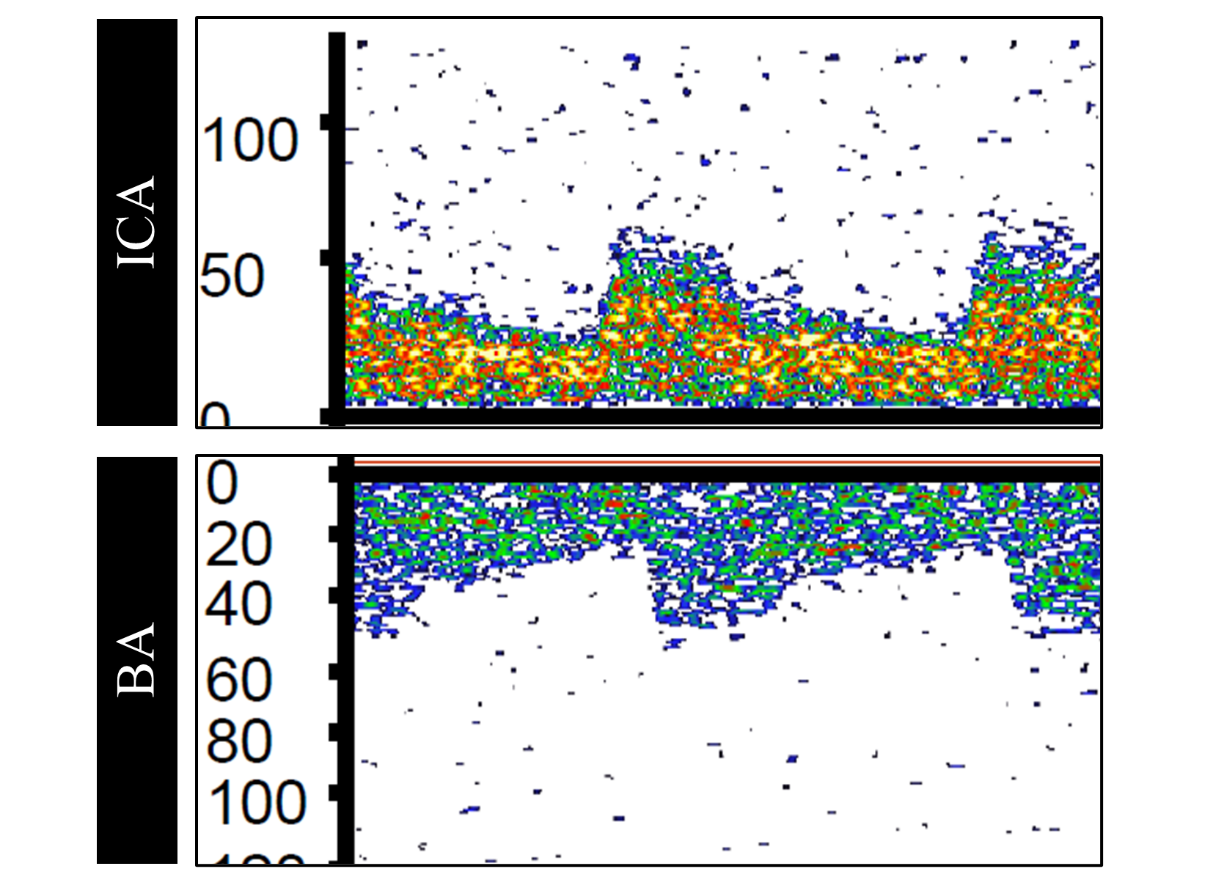
**]**

Abbreviation: ICA, internal carotid artery; BA, basilar artery
